# Analysis of pubertal height gain in post-menarche girls

**DOI:** 10.1097/MD.0000000000047998

**Published:** 2026-03-20

**Authors:** Ja Hyang Cho, Kye Shik Shim

**Affiliations:** a Department of Pediatrics, Kyung Hee University Hospital at Gangdong, Kyung Hee University School of Medicine, Seoul, Korea.

**Keywords:** central precocious puberty, early puberty, final height, gonadotropin-releasing hormone analog treatment

## Abstract

Menarche significantly affects linear growth, and the extent of height gain after menarche remains a key clinical concern. This study aimed to analyze the relationship between menarche timing and pubertal height gain, determine the extent of height gain after menarche, and assess whether treatment is required in cases of early puberty with advanced bone age (BA). A retrospective chart review of 43 patients aged 12 to 16 years with precocious or early puberty was conducted between January and June 2022. Eight patients were excluded due to treatment with recombinant growth hormone. The remaining 35 patients were divided into 2 groups based on whether they received gonadotropin-releasing hormone (GnRH) agonists. Of the 35 patients, 27 (77.1%) were treated with GnRH agonists. The mean age at menarche was delayed (11.5 ± 1.0 vs 12.1 ± 1.7, respectively). The mean predicted adult height (PAH) before menarche was lower in the untreated than in the treated group (156.7 ± 1.8 and 160.0 ± 1.8 cm, respectively, *P* < .05) in the treated group. After menarche, mean PAH was 157.0 ± 0.7 cm in the untreated group and 160.5 ± 0.9 cm in the GnRH agonist group (*P* = .04). Height gain after menarche was greater in the untreated group, 6.6 ± 3.9 versus 4.9 ± 3.0 cm, though it was not statistically significant. Importantly, the final height increase after menarche was negatively correlated with advanced BA before menarche (*r* = −0.092, *P* < .001). The mean age at menarche was delayed in the GnRH agonist group, and the final PAH was higher than that in the untreated group. However, a later age at menarche was associated with lower pubertal height gain. An increase in PAH after treatment can be anticipated in patients with an advanced BA.

## 1. Introduction

Central precocious puberty (CPP) results from premature activation of the hypothalamic–pituitary–gonadal axis before the normal age of pubertal onset.^[1]^ It is defined as the appearance of secondary sexual characteristics before the age of 8 years in girls and 9 years in boys.^[2,3]^ Early puberty may be accompanied by rapid pubertal development, with girls reaching Tanner stage 3 before the age of 9 years, significantly earlier than normal.^[2,3]^ Both early and rapidly progressing puberty lead to premature epiphyseal fusion and short adult height in girls, which may be a characteristic outcome in girls with CPP.

Premature estradiol secretion increases the growth rate and accelerates bone maturation, potentially shortening the growth period and compromising final adult stature.^[4–6]^ Treatment with gonadotropin-releasing hormone (GnRH) agonists suppresses the pituitary-ovarian axis, thereby halting pubertal progression in CPP.^[6]^ Thus, medical treatment results in decreased accelerated growth and bone maturation to the prepubertal stage and eventually improves the final adult height.

The relationship between menarche timing and pubertal growth has been explored for several years, but the effect of treatment on final height (FHt) in girls in early puberty remains controversy.^[7–9]^ Some reports have suggested possible results, such as excessive deceleration of the growth rate, resulting in subsequent worsening of FHt.^[10,11]^ Age at menarche and height gain until FHt, depending on GnRH agonist treatment, may help to understand the course of early puberty or rapidly progressing puberty.

Parents often express concerns that their children’s final adult height will be lower than the target height (TH) after menarche, particularly in cases of precocious puberty.^[1]^ This study aimed to investigate the relationship between menarche timing and final adult height, and to analyze the extent of pubertal height gain after menarche. This study was designed to determine whether delaying menarche alone can result in height gain, and whether early puberty necessitates medical treatment.

## 2. Subjects and methods

### 2.1. Patients

Forty-three girls with suspected early or precocious puberty, who had been followed up until menarche and attended our pediatric endocrinology clinic between January 2022 and June 2022, were initially enrolled in this retrospective case-control study. All patients met the following inclusion criteria: appearance of first pubertal signs (breast buds and/or genital Tanner stage II); plasma luteinizing hormone (LH) response >5 mU/L to GnRH stimulation and plasma estrogen levels appropriate for early puberty; or pubertal pelvic sonographic findings of increased uterine size and ovarian volume^[6,11,12]^; and regular follow-up to completion of puberty. Girls born prematurely or who were small for gestational age, girls with chronic diseases, bone dysplasia, organic brain diseases, congenital adrenal hyperplasia, or other endocrinological abnormalities, and girls who underwent radiation therapy and/or chemotherapy were excluded. Eight patients were excluded due to the use of recombinant growth hormone. Patients with peripheral precocious puberty, including those with exogenous estrogen exposure or chronic systemic steroid use, were excluded. None of the patients presented with neurologic symptoms suggestive of central nervous system pathology. Brain magnetic resonance imaging was selectively performed in 4 patients with rapid pubertal progression, and no abnormal findings were detected. Among the 35 patients, adult height was reported in 6 children (17.1%), which was defined as height at age ≥ 18 years, bone age (BA) ≥ 16 years, and/or a height velocity <1 cm/year.

### 2.2. Treatment

Based on the therapeutic policy at that time, gonadotropin-suppressive therapy was offered to all girls diagnosed with precocious puberty regardless of stature. The patients were divided into 2 groups based on the use of GnRH agonists. Among 35 patients, 27 (77.1%) were treated with GnRH agonists, while the others were included in the study as controls. Therapy was initiated in those who consented at Tanner stage II and consisted of a depo-preparation of the superactive GnRH-analog D-Trp-6-LHRH (Decapeptyl, Ferring Pharmaceuticals Ltd., Saint-Prex, Switzerland) or Leuprorelin Acetate (Leuplin, Takeda Pharmaceuticals Ltd., Tokyo, Japan), administered by injection every 4 week at a calculated dose of 1.5 to 3.0 μg/kg release per day (maximal dose per injection, 3.75 mg) via the subcutaneous or intramuscular route. Therapy was discontinued at chronological age (CA) 11 to 11.5 years, BA 12 to 12.5 years, and/or growth velocity below 4 cm/year.

### 2.3. Follow-up

All girls, both treated and untreated, were regularly followed up at 3-month intervals for height, weight, and pubertal stage. Growth parameters were assessed using Korean National Growth Charts (2017 revision), which are aligned with WHO standards. Growth velocity was derived from annualized height increments using the same Korean reference data. BA was determined every 6 months. All evaluations were performed by the same pediatric endocrinologist and radiologist until puberty reached near adult height, which means that BA was beyond 15 years, and growth velocity was below 2 or 3 cm/year. In this study, we validated hormonal suppression by measuring the peak levels of LH and follicle-stimulating hormone (FSH) at least 1 hour after injection. In all girls, the LH and FSH levels were suppressed throughout the treatment period. Pubertal growth and predicted adult height (PAH) were analyzed before and after menarche.

### 2.4. Methods

Pubertal growth was evaluated and calculated for all the girls before and after menarche.^[13]^ Pubertal staging was performed according to the method described by Marshall and Tanner.^[6,14]^ BA was estimated by standard left-wrist radiography and interpreted using Greulich–Pyle and Bayley–Pinneau.^[13]^ PAH was calculated using the Bayley and Pinneau method with tables for average girls and was found to be more accurate in girls with sexual precocity.^[13]^ The TH was calculated as corrected mid-parental height (MPH) based on the method described by Tanner et al, using the formula: MPH (cm) = [(father’s height + mother’s height)/ 2] − 6.5.^[6]^ Hormonal evaluation included the measurement of basal and GnRH-stimulated LH, FSH, and basal estradiol levels. All hormonal examinations were performed using standard techniques at the endocrine laboratory of our hospital as previously described.^[12]^

### 2.5. Statistical analysis

All analyses were performed using SPSS software (version 24.0; SPSS Inc., Chicago) and R software (version 4.2.3), and the results are expressed as mean ± SD. Differences in auxological data, menarche onset, and predicted final adult height were compared using the Mann–Whitney *U* test. To investigate the association between age at menarche and post-menarche height gain, Spearman correlation analysis was performed for the entire study cohort. Additionally, stratified analyses were conducted within subgroups defined by GnRH agonist treatment status to explore potential treatment-specific associations. Univariate linear regression analyses were employed to identify potential clinical factors associated with pre-menarche PAH (pre-menarche PAH), post-menarche PAH (post-menarche PAH), and pubertal height gain. Subsequently, variables exhibiting a *P*-value <.2 in the univariate models were included in multivariate linear regression models to assess their independent associations with each outcome. Robust standard errors were applied to all the regression models to account for potential heteroscedasticity. The regression results are presented as coefficients with corresponding robust standard errors, 95% confidence intervals, and *P*-values. The BA was determined using the method described by Greulich and Pyle. The PAH was analyzed using BoneXpert version 3.1 (https://bonexpert.com).

## 3. Result

In this study, 43 girls suspected of having early puberty or diagnosed with precocious puberty who had completed GnRH agonist treatment were followed up until menarche. Eight girls (18.8%) who concomitantly received recombinant growth hormone were excluded from the study. Therefore, 35 patients with menarche were included in the study. Of these, 6 (17.1%) were reported to have reached their final adult height. All the participants exhibited pubertal characteristics corresponding to the Tanner stage.

The auxological characteristics of the cohort are summarized in Table 1. At the initial evaluation, there were no significant differences in CA, BA, or Ht between groups. The mean menarche age was delayed in the GnRH agonist-treated group compared to the untreated group (11.5 ± 1.0 vs 12.1 ± 1.7 yr, respectively). At Tanner stage 3, the treated group tended to be younger and had slightly more advanced pubertal development than the untreated group (12.8 ± 0.5 vs 12.9 ± 1.1 yr, respectively). The TH was similar in both groups, with no significant differences between the groups.

**Table 1:** Characteristics of girls with precocious or early puberty who were treated or not treated with GnRH agonist.

	Patients not treated with GnRH agonist	Patients treated with GnRH agonist	*P*-value
	Mean ± SD (Min–Max) or n (%)	Mean ± SD (Min–Max) or n (%)	
Number of patients	8	27	
Initial evaluation			
Chronological age, yr	11.1 ± 1.1 (10.5–12.9)	12.0 ± 0.6 (11.1–13.2)	NS
Bone age, yr	12.9 ± 1.1 (12.5–15)	12.8 ± 0.5 (12.5–13)	NS
Evolution			
Age at menarche, yr	11.2 ± 0.8 (10.5–13.1)	12.1 ± 1.7 (11.7–13.7)	NS
Final evaluation			
Chronological age, yr	12.5 ± 0.5 (10.1–12.9)	13.2 ± 0.5 (12.4–14.7)	NS
Bone age, yr	15.1 ± 0.9 (12.5–15)	14.4 ± 0.9 (13–16)	NS
Growth evolution			
Height at initial evaluation, cm	148.0 ± 6.2 (138.5–157.3)	153.0 ± 5.7 (142.7–168.7)	NS
Height after 1st menstruation, cm	154.6 ± 6.1 (151.3–166.2)	157.9 ± 4.6 (151.6–169.2)	NS
Target height, cm	161.1 ± 3.0 (159.5–162.5)	160.0 ± 3.2 (153.5–171)	NS

GnRH = GnRH = gonadotropin-releasing hormone.

Pubertal height gains after menarche are shown in Table 2. The mean PAH at Tanner stage 3 was lower in the untreated than in the treated group (156.7 ± 1.8 and 160.0 ± 1.8 cm, respectively, *P* = .04). After menarche, mean PAH was 157.0 ± 0.7 cm in the untreated group and 160.5 ± 0.9 cm in the GnRH agonist group (*P* = .04). Finally, when PAH before menarche to TH was compared with PAH after menarche to TH, no significant differences were observed. However, the height gain after menarche was greater in the untreated group 6.6 ± 3.9 versus 4.9 ± 3.0 cm, though it was not statistically significant.

**Table 2:** Pubertal height gain in post-menarche girls.

	Patients not treated with GnRH agonist	Patients treated with GnRH agonist	*P*-value
	Mean ± SD (Min–Max) or n (%)	Mean ± SD (Min–Max) or n (%)	
Number of patients	8	27	
Height at initial evaluation, cm	148.0 ± 6.2	153.0 ± 5.7	.09
Height at 1st menstruation, cm	154.6 ± 6.1	157.9 ± 4.6	.07
Target height, cm	161.1 ± 3.0	160.0 ± 3.2	NS
Predicted adult height at initial evaluation, cm	156.7 ± 5.3	160.0 ± 4.1	.04
Predicted adult height at 1st menstruation, cm	157.0 ± 5.7	160.5 ± 4.5	.04
Growth evolution			
PAH at initial evaluation – target heights, cm	−4.44 ± 4.37	−0.02 ± 4.49	.02
PAH after 1st menstruation–target heights, cm	−4.00 ± 5.00	0 ± 4.63	.02
Pubertal growth gain after menarche, cm	6.6 ± 3.9	4.9 ± 3.0	NS

GnRH = GnRH = gonadotropin-releasing hormone, PAH = predicted adult height.

Multivariate analysis showed that GnRH treatment was associated with a significant increase in PAH in PAH before and after the model (β = 4.03 and β = 4.31, respectively, *P* <.05). In the pubertal growth gain after menarche model, the association between GnRH treatment and growth gain was not significant in univariate analysis (β = −1.64, *P* = .263). However, in the multivariate analysis, a trend toward significance was observed (β = −1.44, *P* = .060) (Table 3).

**Table 3:** Association between clinical variables and predicted adult height before menarche.

Variable	Univariate			Multivariate		
	β (SE)	95% CI	*P*-value	β (SE)	95% CI	*P*-value
GnRH treatment						
Untreated	Ref.			Ref.		
Treated	3.34 (1.96)	[−0.5 to 7.18]	.097	4.03 (1.93)	[0.26–7.81]	.044
Bone age before menarche	1.37 (1.49)	[−1.55 to 4.28]	.365	2.05 (1.24)	[−0.39 to 4.49]	.110
MPH	0.40 (0.23)	[−0.04 to 0.85]	.083	0.62 (0.18)	[0.26–0.98]	.002

CI = confidence interval, GnRH = gonadotropin-releasing hormone, MPH = mid-parental height, SE = standard error.

The association of BA before menarche is shown in Table 4. BA before menarche has emerged as a significant predictor of growth outcome. In both the PAH before menarche and PAH after menarche models, BA did not significantly impact PAH (β = 1.37, *P* = .365 before menarche and β = 1.07, *P* = .476 after menarche in the univariate analysis). However, in the multivariate analysis, the results showed a modest but non-significanttrend in favor of an association (β = 2.05, *P* = .110 for PAH before menarche and β = 1.88, *P* = .136 for PAH after menarche). BA before menarche was a significant predictor of pubertal growth gain after menarche in both univariate (β = −3.29, *P* <.001) and multivariate analyses (β = −3.13, *P* <.001).

**Table 4:** Association between clinical variables and predicted adult height after menarche.

Variable	Univariate			Multivariate		
	β (SE)	95% CI	*P*-value	β (SE)	95% CI	*P*-value
GnRH treatment						
Untreated	Ref.			Ref.		
Treated	3.48 (2.13)	[−0.7 to 7.67]	.112	4.31 (2.03)	[0.33–8.29]	.042
Bone age before menarche	1.07 (1.49)	[−1.84 to 3.98]	.476	1.88 (1.23)	[−0.53 to 4.29]	.136
MPH	0.52 (0.22)	[0.09–0.95]	.024	0.73 (0.19)	[0.36–1.10]	<.001

CI = confidence interval, MPH = mid-parental height, SE = standard error.

MPH consistently emerged as a significant predictor of adult height in both the PAH models. MPH had a moderate and significant effect on the multivariate analysis of PAH before and after menarche (β = 0.62, *P* = .002 vs β = 0.73, *P* <.001, respectively). The relationship between MPH and pubertal growth gain after menarche was not significant in multivariate analysis (β = 0.13, *P* = .184) (Table 5).

**Table 5:** Association between clinical variables and pubertal growth gain after menarche.

Variable	Univariate			Multivariate		
	β (SE)	95% CI	*P*-value	β (SE)	95% CI	*P*-value
GnRH treatment						
Untreated	Ref.			Ref.		
Treated	−1.64 (1.44)	[−4.46 to 1.18]	.263	−1.44 (0.74)	[−2.88 to 0]	.060
Bone age before menarche	−3.29 (0.55)	[−4.37 to −2.21]	<.001	−3.13 (0.48)	[−4.08 to −2.19]	<.001
MPH	0.36 (0.16)	[0.04–0.67]	.032	0.13 (0.1)	[−0.06 to 0.33]	.184

CI = confidence interval, MPH = mid-parental height, SE = standard error.

**Figure 1.**
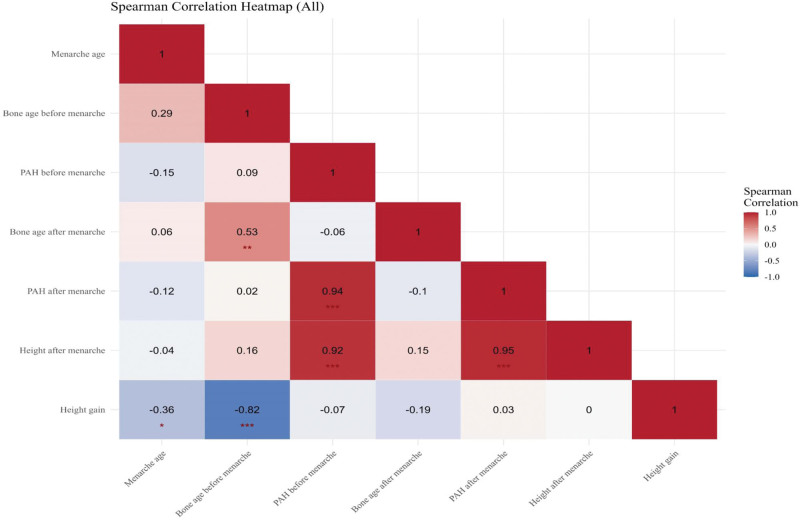
Heatmap of Pearson correlation coefficient matrix. PAH = predicted adult height.

The FHt increased after menarche, showing a strong, statistically significant negative correlation (*r* = −0.92, *P* <.001) (Fig. 1). This result suggests that advanced BA before menarche is associated with a lower height gain after menarche (Fig. 1). Additionally, pubertal height declines over time as skeletal maturation progresses. Therefore, PAH before menarche showed a strong positive correlation with PAH after menarche and height after menarche (0.94 and 0.92, respectively; *P* <.001) (Fig. 1).

## 4. Discussion

The present study investigated the relationship between the timing of menarche and pubertal growth, specifically when menarche occurs in relation to pubertal growth spurt, and how pubertal height gain is associated with the timing of menarche.

Several studies have explored the relationship between age at menarche and the final adult height.^[15]^ Girls who experienced later menarche had a higher final adult height. The estimated pubertal increment of height study revealed that height distribution tended to be 0.31 cm taller every year menarche was delayed.^[7–9,13,15]^ Controversial data on the effects of GnRH agonists on the final adult height have been reported in the literature.^[9,10,16]^ Some studies have demonstrated that GnRH agonists do not lead to a significant final adult height gain.^[9,16,17]^ We found a significant negative correlation between the BA before menarche and height gain.^[9,17]^ This suggests that a more advanced BA before menarche is associated with less height gain after menarche, indicating that the timing of menarche influences the length of the growth period before epiphyseal plate closure.^[9,10,16]^

In this study, we characterized pubertal height outcomes after menarche in girls with suspected early puberty or those diagnosed with precocious puberty and receiving GnRH agonist treatment. The mean age at menarche in the GnRH agonist group was delayed and the predicted final adult height was higher than that in the untreated group. The results indicated that the mean age at menarche was delayed in the GnRH agonist-treated group compared to that in the untreated group. This aligns with previous research findings that GnRH agonist therapy can suppress pubertal progression and postpone menarche, potentially allowing prolonged growth periods.^[9,10,15,16]^ Despite this delay, the pubertal height gain after menarche was not significantly different between the treated and untreated groups. The average gain in height after menarche was greater in girls who menstruated early, in the normal range. By challenging the most common belief that early menarche leads to shorter PAH, healthcare providers can significantly relieve parents’ and children’s anxiety and reduce unnecessary referrals.^[10,15]^ This suggests that while treatment may alter the timing of growth, it does not necessarily lead to a significant increase in final adult height.^[17]^

These results can be understood through underlying physiological mechanisms. The lower pubertal height gain following menarche in treated patients can be understood physiologically. GnRH analog therapy delays menarche and increases PAH primarily through earlier deceleration of bone maturation and restoration toward CA rather than by increasing late-pubertal growth velocity.^[18]^ At the time of menarche, treated patients had more mature skeletal age with higher PAH already achieved; therefore, remaining residual pubertal height gain was smaller, although the final attained height was greater.^[9,18]^

MPH was a significant predictor of adult height in both PAH models. This finding supports the well-established understanding that a child’s genetic potential for height is strongly influenced by the height of their parents.^[8,9,18]^ However, the relationship between MPH and pubertal growth gain after menarche was not significant in multivariate analysis (β = 0.13, *P* = .184). This suggests that. Although MPH can strongly predict overall adult height, it does not necessarily correlate with growth gain during puberty.^[9,18]^

Our analysis also demonstrated that BA before menarche was negatively correlated with post-menarche height gain. BA before menarche was a significant predictor of pubertal growth gain after menarche in both univariate (β = −3.29, *P* <.001) and multivariate analyses (β = −3.13, *P* <.001). This finding implies that girls with an advanced BA at menarche onset may experience less height gain after menarche, which may limit their FHt. Similarly, previous studies suggest that accelerated skeletal maturation can reduce the remaining linear growth potential, particularly in patients during early puberty.^[19,20]^ Even in cases of early puberty, where BA progression is rapid and severe, GnRH agonist administration may improve the expected adult height and can be considered as a treatment option in clinical practice.^[19–21]^ There is considerable variation in pubertal growth, and age at menarche alone may not be a strong predictor of the FHt or height gain.^[9]^ However, this could be an important factor for understanding the different growth patterns of pubertal girls.

Premenstrual estimated PAH was positively correlated with PAH after menarche and height after menarche (*R* = 0.94 and 0.92, respectively; *P* <.001). This finding suggests that hormonal levels before menarche may influence growth during and after puberty, thereby playing a crucial role in final adult height. This aligns with previous studies, indicating that Bayley–Pinneau method predictions may be due to advanced bone maturation in early precocious puberty, likely driven by the stimulatory effect of a slight increase in estrogen on epiphyseal growth plate maturation.^[21]^

Although the untreated group initially had a lower PAH than the treated group at Tanner stage 3, the difference in PAH before and after menarche was not significant. The treated group showed a smaller discrepancy between PAH and TH, suggesting that a predicted height advantage does not always translate into significantly greater final adult height.^[21,22]^ In addition, pubertal height gain was slightly higher in the untreated group; however, the difference was not statistically significant. This underscores the need to assess the long-term benefits of GnRH agonist therapy in terms of height outcomes.^[22]^

This study had some limitations, such as the relatively small sample size, particularly in the subgroup of patients who reached their final adult height. Apart from the sample size, factors such as genetic potential, nutritional status, and environmental influences, which may have influenced the outcomes, have not been extensively analyzed. In addition, as the data were obtained from a single care center, a potential center-specific bias related to referral patterns and treatment practices cannot be excluded. Moreover, because the cohort predominantly consisted of patients of Asian ancestry, the generalizability of our findings to more heterogeneous populations may be limited. Future studies with larger sample sizes and longer follow-up periods are warranted to further clarify the effects of GnRH agonist treatment on the FHt.

In conclusion, although GnRH agonist treatment effectively delays menarche, its impact on FHt remains variable.^[9,23,24]^ BA before menarche appears to be an important determinant of post-menarche height gain, emphasizing the need to carefully consider skeletal maturation in treatment dicision.^[25]^ Additionally, PAH before menarche was a strong predictor of FHt outcomes. Height gain after menarche typically ranges from 4.9 to 6.6 cm, suggesting that excessive concern about an abrupt halt in linear growth may be overstated. With significantly accelerated bone maturation during early puberty, GnRH agonist treatment can be considered to increase PAH, highlighting the potential benefits of interventions in selected patients.^[21]^ However, delaying menarche alone may not necessarily improve the height gain. Given the potential variability in response, individualized treatment strategies are necessary to optimize the growth potential in girls with early or precocious puberty. It is necessary to improve the awareness of parents, especially those who want to suppress menstruation to increase their FHt, as minimal height gain can be achieved after menarche. Further studies on the various factors associated with menarche, age, and height gain are needed.

## Author contributions

**Conceptualization:** Kye Shik Shim.

**Data curation:** Ja Hyang Cho.

**Formal analysis:** Ja Hyang Cho.

**Investigation:** Ja Hyang Cho, Kye Shik Shim.

**Methodology:** Ja Hyang Cho, Kye Shik Shim.

**Project administration:** Ja Hyang Cho, Kye Shik Shim.

**Supervision:** Kye Shik Shim.

**Writing – original draft:** Ja Hyang Cho.

**Writing – review & editing:** Kye Shik Shim.
